# A robust decision making algorithm for handling uncertainty in career planning via a circular intuitionistic fuzzy SWARA WASPAS method

**DOI:** 10.1038/s41598-026-45506-0

**Published:** 2026-04-03

**Authors:** Jinling Chen

**Affiliations:** Publicity Department, Zhejiang Polytechnic University of Mechanical and Electrical Engineering, Zhejiang, 310053 China

**Keywords:** Career planning and employment guidance, Circular intuitionistic fuzzy set, SWARA method, WASPAS method, Multi-attribute group decision-making, Engineering, Mathematics and computing

## Abstract

Uncertainty, incomplete information, and subjective considerations of various experts usually disrupt decision-making in real-life situations, particularly when optimizing vocational training, career planning, and employment guidance schemes. Conventional Stepwise Weight Assessment Ratio Analysis (SWARA) and Weighted Aggregated Sum Product Assessment (WASPAS) models, with their fuzzy counterparts, are based on fixed or point-valued forms and thus are not capable of capturing human hesitations, vagueness, and inconsistency. To mitigate these limitations, this paper presents a hybrid decision-making model that incorporates the use of circular intuitionistic fuzzy set (CIFS) as a part of the SWARA-WASPAS approach. CIFS enables non-membership degrees and membership degrees to be represented as circular regions, acceptably and realistically, of how the experts may articulate uncertainty and hesitation in their evaluations. The CIFS-based SWARA method is used to identify the weights of attributes, and the CIFS-based WASPAS technique is used to evaluate and rank alternatives, using the weighted sum model and weighted product model. The multi-attributes group decision-making (MAGDM) approach proves that the proposed hybrid SWARA- WASPAS method is effective in the context of a hypothetical case study that aims to maximize vocational training, career planning, and employment guidance programs. The findings reveal that “vocational training combined with basic career counseling” always tops the list of all the models. The framework is also stable and robust, as indicated by sensitivity analysis and Spearman rank correlation. The comparative analysis demonstrates that the proposed SWARA–WASPAS method outperforms traditional and fuzzy variants by offering superior uncertainty modeling and greater robustness.

## Introduction

Decision-making is concerned with the process of choosing an appropriate course of action out of a group of available courses of action based on the possible consequences and the trade-offs involved. In practice, this process is frequently complicated since it usually comprises several, and even contradictory, attributes. Multi-attribute decision-making (MADM) is a systematic model of dealing with such complexity, which allows the decision-makers (Ɗ) to evaluate alternatives in terms of a variety of attributes, handle trade-offs, evaluate risks, and achieve overall performance. Consequently, MADM improves the quality and reliability of the decision results. Many scholars have come up with MADM methods in different fields. For instance, Mahmood et al.^1^ applied MADM techniques to medical diagnosis problems under spherical fuzzy set environments. Seikh and Mandal^2^ proposed aggregation operators based on Frank t-norms and t-conorms. Ullah et al.^3^ developed MADM methods using specific aggregation operators to improve decision evaluation. Similarly, Gitinavard et al.^4^ introduced a soft-computing-based approach for hesitant fuzzy complex proportional evaluations, with a particular focus on project safety assessment in the construction industry. Extending the MADM framework, MAGDM incorporates the perspectives of multiple decision-makers when comparing alternatives with several attributes. MAGDM is particularly useful in complex decision-making environments such as management accounting, strategic planning, and sustainability assessment, where the simultaneous consideration of input from various stakeholders and experts is essential. Recently, Wang^5^ proposed an enhanced ARAS-based MAGDM framework to improve decision accuracy. Abbas et al.^6^ integrated MAGDM with the MAIRCA model, while Hussain et al.^7^ introduced a MAGDM approach based on the TOPSIS method. Mousavi et al.^8^ proposed a complex MAGDM technique.

The degree of uncertainty and vagueness is usually high when it comes to complex data modeling and decision-making problems. In an effort to solve this imprecision, Zadeh^9^ formulated initially in 1965 initially formulated the fuzzy set (FS) theory, whereby elements of a classical set are not limited to binary values, but their membership is on a scale of 0 to 1. The conceptual basis allowed a more realistic representation of real-world phenomena in which crisp classification is insufficient. In his work, based on Zadeh, Atanassov^10^ introduced the intuitionistic fuzzy set (IFS) in 1986 as an extension of the FS with the addition of a non-membership function to the membership one. This two-fold representation presents a more descriptive account of uncertainty as it represents at the same time both membership and non-membership levels of an element. In this direction of generalization, the more recent works have involved geometric and circular representations in fuzzy structures. In 2020, Atanassov^11^ proposed a circular intuitionistic fuzzy set (CIFS) where membership and non-membership degrees of every element are modeled as circles and not as a single point in the interpretation space. This circular form allows making a more flexible representation of imprecise values. This coupled setup makes an important contribution to modeling complex, uncertain information in decision-making. Circular fuzzy frameworks have created new areas of research in the broad applicability. As an example, Fahmi et al.^12^ presented Hamacher aggregation operators of MADM problems, which are based on CIFS. Ullah et al.^13^ used Muirhead mean operators under the circular Pythagorean FS to waste material recycling, and Haleemzai et al.^14^ designed a decision-making algorithm of the circular q-rung orthopair fuzzy WASPAS to measure the contribution of the digital economy to the economic growth. Further, Ali and Yang^15^ were able to examine renewable energy systems based on the concept of circular bipolar complex intuitionistic fuzzy set, and Ashraf and Chohan^16^ offered circular spherical fuzzy aggregation operators in the areas of risk evaluation during industrial expansion processes. The Step-Wise Weight Assessment Ratio Analysis (SWARA) technique was proposed by Kersuliene et al.^17^ to calculate the relative weight of and approximate ranking of decision attributes. This method is concerned with the estimation of attribute weights, which is a critical phase that more or less determines the results of the process of decision-making. SWARA is especially dependent on expert judgment,it is more applicable in MAGDM issues where expert judgments are prominent. Its hierarchical nature enables decision-makers to reflect their preferences logically, clearly, and systematically, thus enhancing the clarity and credibility of the weighting process. Besides this, Zavadskas et al.^18^ have suggested the method of the Weighted Aggregated Sum Product Assessment (WASPAS) that combines two popular assessment models, the weighted sum model (WSM) and the weighted product model (WPM). This combination approach assists in a more comprehensive and balanced evaluation, especially in complex decision-making processes that require a number of thought-provoking attributes that are more than could be conflicting through the establishment of many attributes. Building on these foundations, many researchers have further extended the SWARA and WASPAS methods within various fuzzy environments. Dorfeshan et al.^19^ extended the MABAC and WASPAS methods within interval type-2 fuzzy information for aircraft maintenance planning, Bitarafan et al.^20^ introduced a real-time intelligent sensor for structural health monitoring of bridges based on SWARA-WASPAS, while Alam et al.^21^ extended the AHP-WASPAS model to evaluate public cloud computing services. Rani et al.^22^ extended WASPAS within an intuitionistic type-2 fuzzy set framework for multi-attribute physician selection, while Mishra et al.^23^ developed an interval-valued intuitionistic fuzzy WASPAS method. Peng et al.^24^ extended WASPAS, MABAC, and COPRAS methods using hesitant fuzzy soft sets, Karabasevic et al.^25^ applied the SWARA–WASPAS combination to personnel selection problems, whereas Ayyildiz et al.^26^ introduced an AHP–WASPAS approach under Pythagorean fuzzy sets for refugee camp location selection. More recently, Li^27,28^ introduced assessment of management accounting using a decision algorithm based on circular fermatean fuzzy CRITIC and WASPAS methods, Gao et al.^29^ proposed a decision-making algorithm for economic growth assessment based on CRITIC and WASPAS under a circular picture fuzzy framework, whereas Lyu and Zhang^30^ developed a circular Pythagorean fuzzy WASPAS model for physical education decision-making. Vocational training, career planning, and employment guidance optimization have become pressing problems because too many graduates of vocational programs still find themselves unemployed or underemployed despite having technical skills. The conventional vocational training is typically rather narrow ware in terms of acquiring the skills, not sufficiently meeting the needs in terms of career choice, labor market awareness, or job seeking skills. Consequently, the trainees can also learn skills that are not coherent with the present industry requirements, are not certain of the career options they can take, or are not able to adjust well to the workforce. Such an unmet need creates a necessity of incorporating an integrated and optimized strategy that goes beyond mere technical education to encompass planned career planning and focused job placement. The research question upon which the research study has found an answer is whether the optimal and combined model that incorporates vocational training, strategic career planning, and personalized employment counseling can significantly optimize the employment results in comparison to conventional vocational training. The given model focuses on the alignment of training materials with the current real-time labor market demands, assisting trainees to determine the appropriate career goals according to their interests and skills, and providing them with the practical job-finding skills, including how to prepare a resume, interview tactics, and assessment of job-finding flexibility. The model provides career planning and employment advice so that by incorporating career planning and employment advice into the training process, the model is held to assist the trainees in the learning process, in addition to making correct career decisions and actually succeeding in the employment industry. This issue is especially critical since the results of employment depend on a combination of several related factors and not just technical skills. Vocational trainees are usually unable to make the most out of their training due to career indecision, lack of industry exposure, and access to an employment network. The efficacy of the optimization strategies can be evaluated through an experimental comparison of trainees who are subjected to integrated vocational training, career planning, and employment guidance with those who are subjected to conventional training. The findings of this kind of research can inform policy-makers, training systems, and educators on how to re-distribute vocational education systems to be more employment-ready, less competency job mismatch, and more job-ready in the long term. FS theory is especially useful for dealing with these types of problems involving uncertainty, as decision-making processes are often complex and non-linear. To evaluate multidimensional and sometimes conflicting attributes, MAGDM methods provide an effective analytical framework. In recent years, significant progress has been made in this field, with researchers proposing new models and techniques to improve decision-making under ambiguous conditions. For example, Cheng^31^ developed a career planning assistance platform for college students by integrating fuzzy logic with deep learning. Yue^32^ applied a fuzzy control algorithm to personal career planning and entrepreneurship among college students. Li.^27,28^ proposed a method for evaluating college students’ career suitability using a fuzzy clustering algorithm, while El-Sofany et al.^33^ presented an educational and career guidance model based on fuzzy logic principles. Li et al.^34^ adopted the integration of College students’ mental health education and career planning based on feature fuzzy clustering, Xiao et al.^35^ elaborated an optimized technique for college students job searching strategies using fuzzy logic control, while Liu^36^ initiated the optimized technique for college students job searching strategies using fuzzy logic control.

Although SWARA and WASPAS techniques are extensively used in decision-making, the traditional approaches and their fuzzy counterparts are based mostly on fixed or point-value representation. This restricts their ability to reflect the natural hesitation, ambiguity, and variability that are present in the reasoning of human beings. Besides, the current methods fail to be sufficiently malleable to allow the decision-makers to represent subjective trust and uncertainty in terms of membership and non-membership. The instability in the rankings of the MAGDM problems is commonly caused by the diversity and inconsistency of the expert opinions, and cannot be effectively overcome with the traditional approaches. Furthermore, it has not been adequately studied how SWARA and WASPAS can be used as a combined force in a CIFS environment so that their potential as robust and realistic decision-making tools in managing ambiguity and incomplete information can be well exploited.

1. What are the ways of integrating CIFS with SWARA and WASPAS procedures to enhance the model of uncertainty, hesitation, and vagueness when it comes to expert judgments?
2. Is it possible that the hybrid CIFS-based SWARA-WASPAS technique has more plausible and realistic attribute weights and ranks than the traditional fuzzy or crisp decision-making techniques?
3. What is the strength and stability of the rankings generated by the CIFS-based SWARA-WASPAS model in the case of inconsistencies, incompleteness, or ambiguities in the opinions of experts?
4. How well does the CIFS-based hybrid method achieve better decision-making performance in practice, e.g., how can better vocational training/career guidance programs be optimized?

To fulfill the above goal, the investigation will have the following objectives:

1. To apply the SWARA technique to the CIFS setting in computing attributes weights that are indicative of hesitation, vagueness, and subjective uncertainty of experts.
2. To integrate CIFS into the WASPAS model of assessing, rating, and ranking alternatives in a fashion that builds in inconsistencies and unfinished expertise.
3. To implement the hybrid model of CIFS-based SWARA-WASPAS to a real-world example, namely optimization of vocational training, career planning, and employment guidance programs, and to prove the workability of the hybrid model in practice.
4. To determine the robustness and stability of the suggested hybrid model by sensitivity analysis and statistical tests, say the Spearman correlation coefficient.

The proposed manuscript is new in that it combines CIFS with a hybrid SWARA-WASPAS system to derive the MAGDM. The CIFS approach enables the modelling of membership and non-membership degrees as circular regions, unlike the traditional SWARA and WASPAS methods, which use fixed or point-valued representations of fuzzy values. This distinctively describes the human reluctance, ambiguity, and subjectivity of expert decisions, which is a more adaptable and authentic picture of the cognitive processes of the decision-makers. Also, the use of SWARA and WASPAS in the CIFS environment is a novel methodological approach that has not been studied before, and it presents a more holistic solution to managing ambiguity and unavailable information when dealing with complex decision-making issues. The research has a number of contributions. To start with, it offers a powerful system of computing the weights of the attributes that is realistic in terms of expert uncertainty and indecision through the SWARA methodology based on CIFS. Secondly, it improves the assessment and rating of alternatives by the WASPAS method, which is based on the CIFS and takes into consideration inconsistencies and unfinished opinions. Thirdly, the hybrid model is implemented based on the practical case study of the optimization of vocational training, career planning, and employment guidance, revealing its relevance and practicability under the condition of real decision-making. Lastly, the study confirms the stability and reliability of the suggested way by Spearman rank correlation and sensitivity analysis, which gives decision-makers a reliable and scientifically plausible instrument of multi-expert MAGDM in uncertain situations.

This paper is organized as follows. In Section "Preliminaries", a review of CIFS is provided, along with a discussion of aspects related to this article. In Section "Decision-making problem under circular intuitionistic fuzzy SWARA-WASPAS method", an extension of the SWARA-WASPAS methodology in the CIFS environment is proposed. In Section "Case study", the suggested approach is applied to career planning and employment guidance. Robustness and sensitivity are addressed in Section "Sensitivity analysis". In Section “Comparative analysis”, a comparison of the current approaches is provided. Section “Conclusion” contains the study’s conclusion and outlines directions for future research.

## Preliminaries

### Definition 1

Given a fixed universal set $\mathcal{U}$, a circular intuitionistic fuzzy set $A$ within the fixed universal set $\mathcal{U}$ is defined as^11^:

$$A=\left\{\left({\mathcal{M}}_{A}\left(\rm{\chi}\right),{\mathcal{N}}_{A}\left(\rm{\chi}\right);{\mathcal{R}}_{A}\left(\rm{\chi}\right)\right)|\rm{\chi}\in \mathcal{U}\right\}$$

The membership degree is denoted by ${\mathcal{M}}_{A}\left(\chi\right)$, the non-membership degree denoted by ${\mathcal{N}}_{A}\left(\chi\right)$, and the radius of membership and non-membership degrees, denoted by ${\mathcal{R}}_{A}\left(\chi\right)$, with the constraint that:

$0\le {\mathcal{M}}_{A}\left(\chi\right)+{\mathcal{N}}_{A}\left(\chi\right)\le 1$ for all $\chi \in \mathcal{U}$ and ${\mathcal{M}}_{A}\left(\chi\right),{\mathcal{N}}_{A}\left(\chi\right),{\mathcal{R}}_{A}\left(\chi\right)\in \left[\mathrm{0,1}\right]$.

Additionally, the hesitancy degree of CIFS is ${h}_{A}\left(\chi\right)=1-{\mathcal{M}}_{A}\left(\chi\right)-{\mathcal{N}}_{A}\left(\chi\right)$ and the CIF values (CIFVs) represented by ${A}_{J}=({\mathcal{M}}_{{A}_{J}},{\mathcal{N}}_{{A}_{J}},{\mathcal{R}}_{{A}_{J}})$, $\left(J=\mathrm{1,2},\dots ,\mathcal{n}\right)$.

### Definition 2

Let ${A}_{1}=\left({\mathcal{M}}_{1},{\mathcal{N}}_{1},{\mathcal{R}}_{1}\right)$ and ${A}_{2}=\left({\mathcal{M}}_{2},{\mathcal{N}}_{2},{\mathcal{R}}_{2}\right)$ be the two CIFVs, then^37^:

1 $${A}_{1}\oplus{A}_{2}=\begin{array}{c}{\mathcal{M}}_{1}+{\mathcal{M}}_{2}-{\mathcal{M}}_{1}{\mathcal{M}}_{2},{\mathcal{N}}_{1}{\mathcal{N}}_{2},{\mathcal{R}}_{1}{\mathcal{R}}_{2}\end{array}$$

2 $${A}_{1}\oplus{A}_{2}=\left(\begin{array}{c}{\mathcal{M}}_{1}{\mathcal{M}}_{2},{\mathcal{N}}_{1}+{\mathcal{N}}_{2}-{\mathcal{N}}_{1}{\mathcal{N}}_{2},{\mathcal{R}}_{1}{\mathcal{R}}_{2}\end{array}\right)$$

3 $$\partial {A}_{1}=\left(\begin{array}{c}1-{\left(1-{\mathcal{M}}_{1}\right)}^{ \partial },{{\mathcal{N}}_{1}}^{ \partial }, {{\mathcal{R}}_{1}}^{ \partial }\end{array}\right)$$

4 $${\left({A}_{1}\right)}^{ \partial }=\left(\begin{array}{c}{{\mathcal{M}}_{1}}^{ \partial },1-{\left(1-{\mathcal{N}}_{1}\right)}^{ \partial },{{\mathcal{R}}_{1}}^{ \partial }\end{array}\right)$$

5 $${\left({A}_{1}\right)}^{c}=\left\{{\mathcal{N}}_{1},{\mathcal{M}}_{1},{\mathcal{R}}_{1}\right\}$$

### Definition 3

For CIFVs ${A}_{J}\left(J=\mathrm{1,2},3,\dots ,\mathcal{n}\right)$, the CIF weighted geometric (CIFWG) operator is defined as^37^:

$$CIFWG\left({A}_{1},{A}_{2},\dots ,{A}_{J}\right)=\prod_{J=1}^{\mathcal{n}}{{A}_{J}}^{{\omega}_{J}}$$

where the weighted vector $\omega={\left({\omega}_{ 1 },{\omega}_{ 2 },\dots ,{\omega}_{ \mathcal{n} }\right)}^{T}$ of ${A}_{J}\left(J=\mathrm{1,2},3,\dots ,\mathcal{n}\right)$, satisfied the constraint ${\omega}_{J}\ge 0$ and $\sum {\omega}_{J}=1$. Then, the aggregated value of a collection of CIFVs ${A}_{J}\left(J=\mathrm{1,2},3,\dots ,\mathcal{n}\right)$ using the CIFWG operator is also a CIFV, and is defined as:

6 $$CIFWG\left({A}_{1},{A}_{2},\dots ,{A}_{J}\right)=\left(\prod_{J=1}^{\mathcal{n}}{{\mathcal{M}}_{J}}^{{\omega}_{J}},1-\prod_{J=1}^{\mathcal{n}}{\left(1-{\mathcal{N}}_{J}\right)}^{{\omega}_{J}},\prod_{J=1}^{\mathcal{n}}{{\mathcal{R}}_{J}}^{{\omega}_{J}}\right)$$

### Definition 4

Let $A=(\mathcal{M},\mathcal{N},\mathcal{R})$ be the CIFV. Then, $\rm{\breve{S} }$ be the scoring function and $\rm{\breve{A} }$ be the accuracy function of CIFV is defined as^38^:

7 $$\rm{\breve{s}}\left(A\right)=\left(\mathcal{M}-\mathcal{N}\right)\times \mathcal{R}\in \left[-\mathrm{1,1}\right]$$

8 $$\rm{\breve{A} }\left(A\right)=\left(\mathcal{M}+\mathcal{N}\right)\times \mathcal{R}\in \left[\mathrm{0,1}\right]$$

## Decision-making problem under circular intuitionistic fuzzy SWARA-WASPAS method

The combination of the SWARA-WASPAS strategies in the CIFS environment presents a major breakthrough towards the uncertainty in decision-making problems. In practical and real-life decision situations, decision-makers are usually expected to make credible judgments even in the presence of ambiguity, incomplete information, and subjective diversity of expert judgments. Traditional SWARA and WASPAS techniques, together with the available fuzzy extensions, are mainly founded on fixed or point-value fuzzy representations. The identified inadequacies can be successfully dealt with by integrating CIFS into the decision-making model. Under a CIFS setting, membership and non-membership degrees are not defined using precise numerical values; rather, they are represented as circular regions and can fulfill the intuitionistic fuzzy condition. This round-shaped design allows decision-makers to describe their preferences in a more flexible manner and, at the same time, consider hesitation and confidence rates.

The SWARA approach is extended in the CIFS framework, and the determination of the weight of attributes becomes more realistic and credible. Professionals are not limited to providing crisp or definite fuzzy weights but can intuitively express their uncertainty by the use of CIF information. Therefore, the weighted attribute would be a better representation of vagueness and subjectivity in real-world decision-making situations. Equally, the incorporation of CIFS into the WASPAS technique will be very beneficial in the evaluation, scoring, and ranking of alternatives. Even though WASPAS is already characterized by a good performance of ranking due to the use of the WSM and WPM, the extension of the CIFS environment explicitly includes hesitations, uncertainty, and inconsistencies in judgments made by the experts. This not only makes the rankings more reliable but also more stable, especially when the opinions of the experts are not accurate, incomplete, or contradictory.

### Methodology of the MAGDM problem

**Fig. 1:**
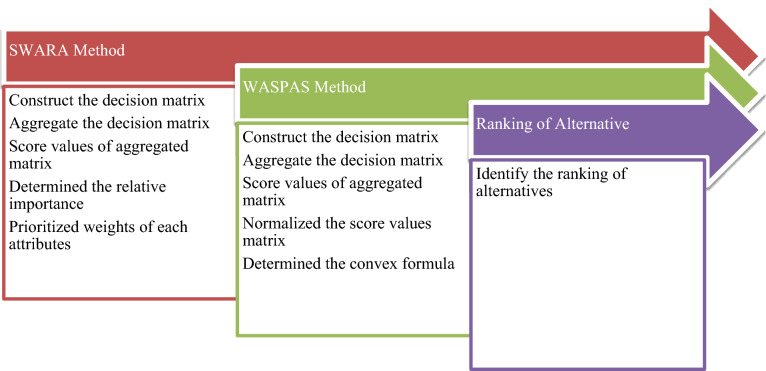
Evaluation of the MAGDM matter through the SWARA-WASPAS method.

The suggested hybrid structure that is based on CIFS provides a methodology for the MAGDM issues, which are unclear and ambiguous. The general pattern of the suggested approach is depicted in Fig. 1, and the specific stages of the hybrid SWARA-WASPAS model in the CIFS environment are given below:

**Step 1:** Use the CIFS linguistic terms shown in Table 1 to determine the weights of decision-makers. Here, we utilized the scoring function from Eq. (7) first and then applied Eq. (9).

**Table 1:** For the evaluation of the case study, these linguistic terms.

Linguistic term	$\mathcal{M}$	$\mathcal{N}$	$\mathcal{R}$
Superior (SUP)	0.95	0.05	0.50
Very high (VH)	0.80	0.15	0.48
Significant (SFT)	0.65	0.25	0.45
Extensive (EXT)	0.50	0.35	0.43
Considerable (CON)	0.40	0.45	0.43
Medium (MDM)	0.30	0.55	0.43
Nominal (NOM)	0.25	0.65	0.45
Very low (VL)	0.20	0.75	0.48
Almost none (ALN)	0.15	0.85	0.50

9 $$\frac{{\sum }_{k}\rm{\breve{S}}\left({{\chi}^{k}}_{\mathrm{i} J}\right)}{{\sum }_{k}^{r}\left({\sum }_{k}\rm{\breve{S}}\left({{\chi}^{k}}_{\mathrm{i} J}\right)\right)}$$

**Step 2:** Decision-makers utilized the linguistic terms from Table 1 to assess the weights of each attribute, ${\mathcal{D}}_{k}\left(k=\mathrm{1,2},3,\dots ,r\right)$. Drawing from these evaluations, the personal decision matrix is formulated in Eq. (10):

10 $${M}_{r\times \mathcal{n}}=\begin{array}{c}{\mathcal{D}}_{1}\\ {\mathcal{D}}_{2}\\ \vdots \\ {\mathcal{D}}_{r}\end{array} \left[\begin{array}{cccc}{\mathcal{C}}_{1}& {\mathcal{C}}_{2}& \dots & {\mathcal{C}}_{\mathcal{n}}\\ {\chi}_{11}& {\chi}_{12}& \dots & {\chi}_{1\mathcal{n}}\\ {\chi}_{21}& {\chi}_{22}& \dots & {\chi}_{2\mathcal{n}}\\ \vdots & \vdots & \ddots & \vdots \\ {\chi}_{r1}& {\chi}_{r2}& \dots & {\chi}_{r\mathcal{n}}\end{array}\right]$$

**Step 3:** The decision matrix referenced in the equation that follows: The weights of the decision-makers are incorporated into (10), and aggregation is performed using the CIFWG operators as specified in Eq. (6).

**Step 4:** The score value of each attribute is derived from the aggregated decision matrix, as indicated by the equation below (7). The attribute scores are then ranked from highest to lowest.

### Step 5: Circular intuitionistic fuzzy SWARA technique

**Step 5.1:** Beginning with the second characteristic, the decision makers assess the relative values of each attribute in comparison to the one that came before it, as demonstrated in Eq. (11). The relative significance of each attribute is outlined below.

11 $${\mathcal{S}}_{\mathrm{J}}=\left\{\begin{array}{c}{\mathcal{S}}_{1}=0 \\ {\mathcal{S}}_{\mathrm{J}}\text{ compare }{\mathcal{C}}_{\mathrm{J}}\text{ to }{\mathcal{C}}_{\mathrm{J}-1}\end{array}\right.$$

**Step 5.2:** The coefficient shows that the relative significance of the next attribute decreases by a specific percentage as outlined in Eq. (12).

12 $${\kappa }_{\mathrm{J}}=\left\{\begin{array}{c}1 \text{ if J}=1\\ {\mathcal{S}}_{\mathrm{J}}+1 \text{if J}>1\end{array}\right.$$

**Step 5.3:** The recalculated weight for each attribute clarifies its relative weights before normalization. The preceding weight is divided by the corresponding coefficient to obtain each weight, as shown in Eq. (13).

13 $${\rho }_{\mathrm{J}}=\left\{\begin{array}{c}1 \text{ if J}=1\\ \frac{{\kappa }_{\mathrm{J}-1}}{{\kappa }_{\mathrm{J}}} \text{if J}>1\end{array}\right.$$

**Step 5.4:** In the final step, the weights that have been recalculated are normalized to ensure that the sum of all attribute weights equals one. These normalized values presented in Eq. (14) correspond to the final priority weights of the attributes.

14 $${\omega}_{J}=\frac{{\rho }_{J}}{{\sum }_{J=1}^{\mathcal{n}}{\rho }_{J}}$$

### Step 6: Circular intuitionistic fuzzy WASPAS technique

**Step 6.1:** The decision-makers evaluate each alternative for each attribute using the linguistic terms provided in Table 1. The decision matrix under examination is constructed as depicted in Eq. (15):

15 $${{M}^{k}}_{\mathcal{m}\times \mathcal{n}}=\begin{array}{c}{\mathcal{A}}_{1}\\ {\mathcal{A}}_{2}\\ \vdots \\ {\mathcal{A}}_{\mathcal{m}}\end{array} \left[\begin{array}{cccc}{\mathcal{C}}_{1}& {\mathcal{C}}_{2}& \dots & {\mathcal{C}}_{\mathcal{n}}\\ {{\chi}^{k}}_{11}& {{\chi}^{k}}_{12}& \dots & {{\chi}^{k}}_{1\mathcal{n}}\\ {{\chi}^{k}}_{21}& {{\chi}^{k}}_{22}& \dots & {{\chi}^{k}}_{2\mathcal{n}}\\ \vdots & \vdots & \ddots & \vdots \\ {{\chi}^{k}}_{\mathcal{m}1}& {{\chi}^{k}}_{\mathcal{m}2}& \dots & {{\chi}^{k}}_{\mathcal{m}\mathcal{n}}\end{array}\right]$$

where ${{\chi}^{k}}_{\mathrm{i} J}=\left({{\mathcal{M}}^{k}}_{\mathrm{i} J},{{\mathcal{N}}^{k}}_{\mathrm{i} J},{{\mathcal{R}}^{k}}_{\mathrm{i} J}\right)$ be the CIFFV represents an alternative ${\mathcal{A}}_{\mathrm{i} }\left(\mathrm{i} =\mathrm{1,2},3,\dots ,\mathcal{m}\right)$ with respect to the attribute ${\mathcal{C}}_{J}\left(J=\mathrm{1,2},3,\dots ,\mathcal{n}\right)$.

**Step 6.2:** The decision matrix shown in Eq. (15) is solidified with the CPyFWG operators outlined in Eq. (6), considering the weights of the attributes as well as those of the decision-makers. The resulting aggregated decision matrix is displayed in Eq. (16).

16 $${\mathrm{M}}_{\mathcal{m}\times \mathcal{n}}=\begin{array}{c}{\mathcal{A}}_{1}\\ {\mathcal{A}}_{2}\\ \vdots \\ {\mathcal{A}}_{\mathcal{m}}\end{array} \left[\begin{array}{cccc}{\mathcal{C}}_{1}& {\mathcal{C}}_{2}& \dots & {\mathcal{C}}_{\mathcal{n}}\\ {\rm{\chi}}_{11}& {\rm{\chi}}_{12}& \dots & {\rm{\chi}}_{1\mathcal{n}}\\ {\rm{\chi}}_{21}& {\rm{\chi}}_{22}& \dots & {\rm{\chi}}_{2\mathcal{n}}\\ \vdots & \vdots & \ddots & \vdots \\ {\rm{\chi}}_{\mathcal{m}1}& {\rm{\chi}}_{\mathcal{m}2}& \dots & {\rm{\chi}}_{\mathcal{m}\mathcal{n}}\end{array}\right]$$

**Step 6.3:** The score for each attribute is calculated using Eq. (7) from the aggregated decision matrix shown in Eq. (16).

**Step 6.4:** The attribute scores are normalized using Eq. (17) to convert all values to a common scale. Depending on whether the attribute represents a benefit or a cost, normalization rules are utilized.

17 $$\aleph \rm{\breve{S}}\left({\chi}_{\mathrm{i} J}\right)=\left\{\begin{array}{c}\frac{\rm{\breve{S}}\left({\chi}_{\mathrm{i} J}\right)}{\underset{ \mathrm{i} }{\mathrm{max}}\left(\rm{\breve{S}}\left({\chi}_{\mathrm{i} J}\right)\right)}; \text{for benefit attribute} \\ \frac{\rm{\breve{S}}\left({\chi}_{\mathrm{i} J}\right)}{\underset{ \mathrm{i} }{\mathrm{min}}\left(\rm{\breve{S}}\left({\chi}_{\mathrm{i} J}\right)\right)}; \text{for cost attribute}\end{array}\right.$$

**Step 6.5:** The first evaluation of the alternatives using the WASPAS method is done with the additive model, represented by ${Q}_{\mathrm{i} }^{1}$, as shown in Eq. (18).

18 $${Q}_{\mathrm{i} }^{1}=\sum_{J=1}^{\mathcal{n}}\aleph {\rm{\breve{S}}}\left({\chi}_{\mathrm{i} J}\right){\omega}_{J}$$

**Step 6.6:** Use the multiplicative model, indicated by ${Q}_{\mathrm{i} }^{2}$ and illustrated in Eq. (19), to assess the alternatives.

19 $${Q}_{\mathrm{i} }^{2}=\prod_{J=1}^{\mathcal{n}}{\aleph \rm{\breve{S}}\left({\chi}_{\mathrm{i} J}\right)}^{{\omega}_{J}}$$

**Step 6.7:** The WASPAS method enhances ranking accuracy through a convex combination of the WSM and WPM. The overall performance score ${Q}_{\mathrm{i} }$ is determined using Eq. (20), where $\lambda \in \left[\mathrm{0,1}\right]$.

20 $${Q}_{\mathrm{i} }=\lambda {Q}_{\mathrm{i} }^{1}+\left(1-\lambda \right){Q}_{\mathrm{i} }^{2}$$

**Step 7:** Ultimately, the option with the highest total score is identified as the best choice. The alternatives are ranked based on their respective score values, with higher scores indicating better performance.

## Case study

In order to prove the usefulness and efficiency of the suggested hybrid SWARA-WASPAS approach in the framework of the CIFS, a case study was performed in the framework of optimizing vocational training, career planning, and employment guidance programs. The case study is about a vocational training institute that seeks to improve employment results by choosing the best training-guidance approach in cases of uncertainty, imprecision, and subjective expert information.

Given the qualitative nature of these attributes and the inherent uncertainty in expert assessments, the CIFS framework is employed to capture both membership and non-membership degrees along with their circular uncertainty boundaries. A panel of experts consisting of vocational trainers, career counselors, and industry representatives provided their evaluations using linguistic terms, which were then transformed into CIFVs. This approach allows a more realistic representation of evaluating the strategies of optimizing vocational training, career planning, and employment guidance.

In the case study, the decision issue is to compare and prioritize various alternative vocational development strategies that are aimed at enhancing the employability of trainees. Five options are discussed: traditional vocational training (${\mathcal{A}}_{1}$), vocational training combined with basic career counseling (${\mathcal{A}}_{2}$), industry-aligned vocational training with structured career planning (${\mathcal{A}}_{3}$), vocational training integrated with personalized career planning and job-search skill development (${\mathcal{A}}_{4}$), fully optimized model combining industry-aligned vocational training, individualized career planning, continuous employment guidance, and employer partnerships (${\mathcal{A}}_{5}$). These options are assessed on several indicators representing the dimensions of program effectiveness that are essential, such as skill–job alignment (${\mathcal{C}}_{\boldsymbol{ }1}$), career clarity (${\mathcal{C}}_{\boldsymbol{ }2}$), employment readiness (${\mathcal{C}}_{\boldsymbol{ }3}$), placement rate (${\mathcal{C}}_{\boldsymbol{ }4}$), job sustainability (${\mathcal{C}}_{\boldsymbol{ }5}$). The primary objective of this hypothetical case study was to develop a robust methodological framework capable of handling uncertainty, hesitation, and subjective expert judgments in MAGDM problems.

### Analyzed the methodology of the MAGDM approach

The execution of the proposed MAGDM algorithm is carried out step by step, as detailed by the mathematical formulations in Section "Decision-making problem under circular intuitionistic fuzzy SWARA-WASPAS method".

**Step 1:** The significance weights for the three decision experts were established using the linguistic terms outlined in Table 1 and by applying Eq. (9), as detailed in Table 2.

**Table 2:** The weights of decision-makers used CIFS linguistic terms.

Group of decision-makers	Vocational trainers	Career counselor	Industry representative	Weights
${\mathcal{D}}_{1}$	EXT	SUP	EXT	0.39
${\mathcal{D}}_{2}$	SFT	MDM	SUP	0.35
${\mathcal{D}}_{3}$	MDM	VH	SFT	0.26

**Step 2:** Three evaluated the attribute weights, using the linguistic terms listed in Table 1. The resulting decision matrix is presented in Table 3.

**Table 3:** The weights of attributes used CIFS linguistic terms.

**Group of decision-makers**	${\mathcal{C}}_{1}$	${\mathcal{C}}_{2}$	${\mathcal{C}}_{3}$	${\mathcal{C}}_{4}$	${\mathcal{C}}_{5}$
${\mathcal{D}}_{1}$	SFT	CON	EXT	EXT	SFT
${\mathcal{D}}_{2}$	VH	EXT	SFT	SUP	VH
${\mathcal{D}}_{2}$	SUP	VH	VH	CON	EXT

**Step 3:** The decision matrix shown in Table 3 was aggregated using the CIFWG operator as specified in Eq. (6), while considering the weights of the decision-makers. The aggregated decision matrix that was created is shown in Table 4.

**Table 4:** Aggregated decision matrix.

Attributes	Aggregated values
${\mathcal{C}}_{1}$	$$\left(\begin{array}{c}\mathrm{0.77,0.17,0.47}\end{array}\right)$$
${\mathcal{C}}_{2}$	$$\left(\begin{array}{c}\mathrm{0.52,0.35,0.44}\end{array}\right)$$
${\mathcal{C}}_{3}$	$$\left(\begin{array}{c}\mathrm{0.62,0.27,0.45}\end{array}\right)$$
${\mathcal{C}}_{4}$	$$\left(\begin{array}{c}\mathrm{0.59,0.29,0.45}\end{array}\right)$$
${\mathcal{C}}_{5}$	$$\left(\begin{array}{c}\mathrm{0.65,0.25,0.45}\end{array}\right)$$

**Step 4:** The score values in the aggregated decision matrix presented in Table 4 were derived using Eq. (7). These score values, which are ranked from highest to lowest, can be found in Table 5.

**Table 5:** Score values inform the descending order.

Attributes	Score values
${\mathcal{C}}_{1}$	0.29
${\mathcal{C}}_{5}$	0.18
${\mathcal{C}}_{3}$	0.16
${\mathcal{C}}_{4}$	0.14
${\mathcal{C}}_{2}$	0.07

## Step 5: Used circular intuitionistic fuzzy SWARA technique

**Step 5.1:** The relative significance of each attribute is assessed using Eq. (11) and presented in Table 6.

**Table 6:** Relative importance matrix.

**Attributes**	${\mathcal{S}}_{\mathrm{J}}$
${\mathcal{C}}_{1}$	0.00
${\mathcal{C}}_{5}$	0.11
${\mathcal{C}}_{3}$	0.02
${\mathcal{C}}_{4}$	0.02
${\mathcal{C}}_{2}$	0.07

**Step 5.2:** Using Eq. (12), the adjustment coefficient is determined, which reduces the relative importance of each subsequent attribute. The computed coefficients can be found in Table 7.

**Table 7:** Adjustment coefficients matrix.

**Attributes**	${{{\kappa}}}_{\mathrm{J}}$
${\mathcal{C}}_{1}$	1.00
${\mathcal{C}}_{5}$	1.11
${\mathcal{C}}_{3}$	1.02
${\mathcal{C}}_{4}$	1.02
${\mathcal{C}}_{2}$	1.07

**Step 5.3:** The values indicating the relative importance of each attribute before normalization, which have been recalculated for weight normalization, are derived using Eq. (13). These values are found in Table 8.

**Table 8:** Attributes recalculated weights.

**Attributes**	${{{\rho}}}_{\mathrm{J}}$
${\mathcal{C}}_{1}$	1.00
${\mathcal{C}}_{5}$	0.90
${\mathcal{C}}_{3}$	1.09
${\mathcal{C}}_{4}$	1.00
${\mathcal{C}}_{2}$	0.95

**Step 5.4:** Finally, the weights for all attributes are determined by normalizing the recalculated weights using Eq. (14). The normalized weights resulting from this are shown in Table 9.

**Table 9:** Attributes weights.

**Attributes**	${\omega}_{J}$
${\mathcal{C}}_{1}$	0.21
${\mathcal{C}}_{5}$	0.18
${\mathcal{C}}_{3}$	0.22
${\mathcal{C}}_{4}$	0.20
${\mathcal{C}}_{2}$	0.19

## Step 6: Used circular intuitionistic fuzzy WASPAS technique

**Step 6.1:** The three individuals responsible for making decisions each developed their own decision matrix, based on the linguistic terms outlined in Table 1. The alternatives’ aggregated linguistic assessments regarding each attribute are shown in Table 10.

**Table 10:** Utilized CIF linguistic terms for alternative assessment.

$\mathcal{D}$	$\mathcal{A}/\mathcal{C}$	${\mathcal{C}}_{1}$	${\mathcal{C}}_{2}$	${\mathcal{C}}_{3}$	${\mathcal{C}}_{4}$	${\mathcal{C}}_{5}$
${\mathcal{D}}_{1}$	${\mathcal{A}}_{1}$	VH	CON	SFT	SFT	EXT
	${\mathcal{A}}_{2}$	EXT	VH	VH	VH	VH
	${\mathcal{A}}_{3}$	SFT	EXT	EXT	SUP	SUP
	${\mathcal{A}}_{4}$	EXT	SUP	SUP	CON	SFT
	${\mathcal{A}}_{5}$	VH	EXT	SFT	EXT	EXT
${\mathcal{D}}_{2}$	${\mathcal{A}}_{1}$	VL	SFT	SFT	EXT	SFT
	${\mathcal{A}}_{2}$	SFT	VH	SUP	VH	VH
	${\mathcal{A}}_{3}$	VH	EXT	EXT	ALN	SUP
	${\mathcal{A}}_{4}$	SFT	SUP	VH	VH	CON
	${\mathcal{A}}_{5}$	VH	SFT	CON	VH	EXT
${\mathcal{D}}_{3}$	${\mathcal{A}}_{1}$	EXT	SFT	CON	EXT	EXT
	${\mathcal{A}}_{2}$	SUP	SUP	VH	VH	VH
	${\mathcal{A}}_{3}$	SFT	EXT	EXT	SUP	ALN
	${\mathcal{A}}_{4}$	MDM	VH	SUP	SFT	VH
	${\mathcal{A}}_{5}$	SFT	CON	EXT	EXT	VH

**Step 6.2:** The individual decision matrix shown in Table 10 is merged using the CIFWG operator defined in Eq. (6), considering the weights of both the decision-makers and the attributes. The aggregated decision matrix that resulted is shown in Table 11.

**Table 11:** Aggregated decision matrix.

$\mathcal{A}/\mathcal{C}$	${\mathcal{C}}_{1}$	${\mathcal{C}}_{2}$	${\mathcal{C}}_{3}$	${\mathcal{C}}_{4}$	${\mathcal{C}}_{5}$
${\mathcal{A}}_{1}$	$$\left(\begin{array}{c}\mathrm{0.57,0.34,0.46}\end{array}\right)$$	$$\left(\begin{array}{c}\mathrm{0.49,0.37,0.44}\end{array}\right)$$	$$\left(\begin{array}{c}\mathrm{0.57,0.31,0.44}\end{array}\right)$$	$$\left(\begin{array}{c}\mathrm{0.62,0.26,0.45}\end{array}\right)$$	$$\left(\begin{array}{c}\mathrm{0.59,0.29,0.44}\end{array}\right)$$
${\mathcal{A}}_{2}$	$$\left(\begin{array}{c}\mathrm{0.62,0.26,0}.\end{array}45\right)$$	$$\left(\begin{array}{c}\mathrm{0.84,0.12,0.48}\end{array}\right)$$	$$\left(\begin{array}{c}\mathrm{0.82,0.14,0.48}\end{array}\right)$$	$$\left(\begin{array}{c}\mathrm{0.78,0.17,0}.\end{array}47\right)$$	$$\left(\begin{array}{c}\mathrm{0.76,0.18,0}.\end{array}47\right)$$
${\mathcal{A}}_{3}$	$$\left(\begin{array}{c}\mathrm{0.67,0.24,0}.\end{array}45\right)$$	$$\left(\begin{array}{c}\mathrm{0.50,0.35,0.43}\end{array}\right)$$	$$\left(\begin{array}{c}\mathrm{0.50,0.35,0.43}\end{array}\right)$$	$$\left(\begin{array}{c}\mathrm{0.72,0.28,0}.\end{array}50\right)$$	$$\left(\begin{array}{c}\mathrm{0.58,0.42,0.50}\end{array}\right)$$
${\mathcal{A}}_{4}$	$$\left(\begin{array}{c}\mathrm{0.45,0.40,0}.\end{array}43\right)$$	$$\left(\begin{array}{c}\mathrm{0.91,0.08,0.49}\end{array}\right)$$	$$\left(\begin{array}{c}\mathrm{0.93,0.07,0}.\end{array}50\right)$$	$$\left(\begin{array}{c}\mathrm{0.51,0.36,0}.\end{array}44\right)$$	$$\left(\begin{array}{c}\mathrm{0.64,0.26,0}.\end{array}45\right)$$
${\mathcal{A}}_{5}$	$$\left(\begin{array}{c}\mathrm{0.76,0.18,0}.\end{array}47\right)$$	$$\left(\begin{array}{c}\mathrm{0.49,0.37,0.43}\end{array}\right)$$	$$\left(\begin{array}{c}\mathrm{0.56,0.31,0}.\end{array}44\right)$$	$$\left(\begin{array}{c}\mathrm{0.54,0.32,0}.\end{array}43\right)$$	$$\left(\begin{array}{c}\mathrm{0.57,0.30,0.44}\end{array}\right)$$

**Step 6.3:** In Step 6.3, the score values for each attribute in the aggregated decision matrix (Table 11) are calculated using Eq. (7). These score values are summarized in Table 12.

**Table 12:** Score values decision matrix.

$\mathcal{A}/\mathcal{C}$	${\mathcal{C}}_{1}$	${\mathcal{C}}_{2}$	${\mathcal{C}}_{3}$	${\mathcal{C}}_{4}$	${\mathcal{C}}_{5}$
${\mathcal{A}}_{1}$	0.11	0.05	0.12	0.16	0.13
${\mathcal{A}}_{2}$	0.16	0.34	0.33	0.29	0.27
${\mathcal{A}}_{3}$	0.20	0.06	0.06	0.22	0.08
${\mathcal{A}}_{4}$	0.02	0.41	0.43	0.06	0.17
${\mathcal{A}}_{5}$	0.27	0.05	0.11	0.09	0.12

**Step 6.4:** The score values displayed in Table 12 are standardized using Eq. (17) to align them on a common scale. The normalized decision matrix that resulted is shown in Table 13.

**Table 13:** Normalized matrix.

$\mathcal{A}/\mathcal{C}$	${\mathcal{C}}_{1}$	${\mathcal{C}}_{2}$	${\mathcal{C}}_{3}$	${\mathcal{C}}_{4}$	${\mathcal{C}}_{5}$
${\mathcal{A}}_{1}$	0.39	0.12	0.27	0.56	0.50
${\mathcal{A}}_{2}$	0.59	0.84	0.77	1.00	1.00
${\mathcal{A}}_{3}$	0.73	0.16	0.15	0.77	0.29
${\mathcal{A}}_{4}$	0.09	1.00	1.00	0.22	0.64
${\mathcal{A}}_{5}$	1.00	0.13	0.26	0.32	0.43

**Step 6.5 to Step 6.7:** The additive, multiplicative, and SWARA-WASPAS models outlined in Eq. (18), Eq. (19), and Eq. (20) are used to combine the normalized decision matrix shown in Table 13, applying the attribute weights obtained from Table 9. The performance scores for ${Q}_{\mathrm{i} }^{1}$, ${Q}_{\mathrm{i} }^{2}$, and ${Q}_{\mathrm{i} }$ are shown in Table 14.

**Table 14:** Proposed hybrid models.

**Alternatives**	${{{Q}}}_{\mathrm{i} }^{1}$	${{{Q}}}_{\mathrm{i} }^{2}$	${{{Q}}}_{\mathrm{i} }$
${\mathcal{A}}_{1}$	0.37	0.33	0.35
${\mathcal{A}}_{2}$	0.83	0.82	0.83
${\mathcal{A}}_{3}$	0.42	0.32	0.38
${\mathcal{A}}_{4}$	0.58	0.41	0.49
${\mathcal{A}}_{5}$	0.44	0.35	0.39

**Step 7:** An alternative ${\mathcal{A}}_{2}$, denoting “vocational training combined with basic career counseling,” attains the highest score values in each model. The ranking results and corresponding score values for all alternatives are displayed in Table 15.

**Table 15:** Score values and ranking.

**Proposed**	${\mathcal{A}}_{1}$	${\mathcal{A}}_{2}$	${\mathcal{A}}_{3}$	${\mathcal{A}}_{4}$	${\mathcal{A}}_{5}$	**Ranking result**	**Best**
${{{Q}}}_{\mathrm{i} }^{1}$	0.37	0.83	0.42	0.58	0.44	${\mathcal{A}}_{2}>{\mathcal{A}}_{4}>{\mathcal{A}}_{5}>{\mathcal{A}}_{3}>{\mathcal{A}}_{1}$	${\mathcal{A}}_{2}$
${{{Q}}}_{\mathrm{i} }^{2}$	0.33	0.82	0.32	0.41	0.35	${\mathcal{A}}_{2}>{\mathcal{A}}_{4}>{\mathcal{A}}_{5}>{\mathcal{A}}_{1}>{\mathcal{A}}_{3}$	${\mathcal{A}}_{2}$
${{{Q}}}_{\mathrm{i} }$	0.35	0.83	0.38	0.49	0.39	${\mathcal{A}}_{2}>{\mathcal{A}}_{4}>{\mathcal{A}}_{5}>{\mathcal{A}}_{3}>{\mathcal{A}}_{1}$	${\mathcal{A}}_{2}$

**Fig. 2:**
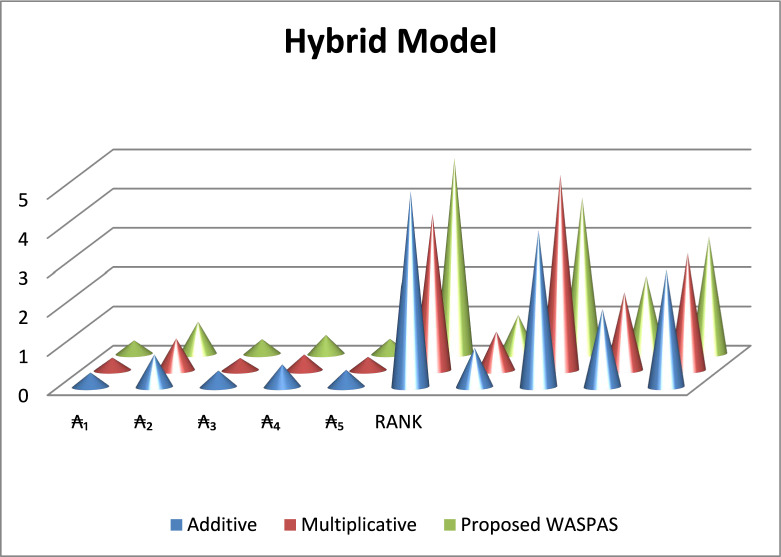
Illustration of the alternative that follows from the work commenced.

These findings confirm the robustness of the proposed approach, as an alternative ${\mathcal{A}}_{2}$ consistently occupies the top position across the additive model, multiplicative model, and combined WASPAS model. Furthermore, Fig. 2 illustrates the ranking patterns and score distributions produced by the additive model, multiplicative model, and SWARA-WASPAS method, providing a distinct visual comparison of the alternatives’ performance.

### Spearman rank correlation coefficient

This subsection employs Spearman Rank Correlation Coefficient^39^ to statistically assess the consistency and robustness of the established additive, multiplicative, and WASPAS ranking model. The analysis treats the five alternatives as ranking objects and the three decision-making methods as judges. A high Spearman rank correlation ($\rho$ close to 1) indicates that the rankings are stable and robust across different models.

**Step 1:** Using Table 15, the ranking of the five alternatives under each model is identified, based on the results shown in Table 16.

**Table 16:** Each model alternative ranking.

**Alternatives**	${{{Q}}}_{\mathrm{i} }^{1}$	${{{Q}}}_{\mathrm{i} }^{2}$	${{{Q}}}_{\mathrm{i} }$
${\mathcal{A}}_{1}$	5	4	5
${\mathcal{A}}_{2}$	1	1	1
${\mathcal{A}}_{3}$	4	5	4
${\mathcal{A}}_{4}$	2	2	2
${\mathcal{A}}_{5}$	3	3	3

**Step 2:** The number of alternatives indicated by $\mathcal{n}=5$ and the difference between the ranks of each alternative in the two models are indicated by ${d}_{\mathrm{i} }$. The Spearman rank correlation coefficient can be computed pairwise between any two models and is shown in Eq. (21):

21 $$\rho =1-\frac{6\sum {d}_{\mathrm{i} }^{2}}{\mathcal{n}\left({\mathcal{n}}^{2}-1\right)}$$

**Step 3:** Used Eq. (21) to check the robustness between ${Q}_{\mathrm{i} }^{1}$ and ${Q}_{\mathrm{i} }^{2}$ the difference result is displayed in Table 17.

**Table 17:** Ranking difference.

**Alternatives**	${{{Q}}}_{\mathrm{i} }^{1}$	${{{Q}}}_{\mathrm{i} }^{2}$	${{{d}}}_{\mathrm{i} }$	${{{d}}}_{\mathrm{i} }^{2}$
${\mathcal{A}}_{1}$	5	4	1	1
${\mathcal{A}}_{2}$	1	1	0	0
${\mathcal{A}}_{3}$	4	5	−1	1
${\mathcal{A}}_{4}$	2	2	0	0
${\mathcal{A}}_{5}$	3	3	0	0

$$\rho =1-\frac{6\left(2\right)}{5\left(25-1\right)}=1-\frac{12}{120}=1-0.1=0.9$$

This implies that the Spearman correlation between these two models is very strong. In a similar way, the Spearman correlation between ${{{Q}}}_{\boldsymbol{\mathrm{i} }}^{2}$ and ${{{Q}}}_{\boldsymbol{\mathrm{i} }}$ is 0.9, and between ${{{Q}}}_{\boldsymbol{\mathrm{i} }}$ and ${{{Q}}}_{\boldsymbol{\mathrm{i} }}^{1}$ is 1. This confirms high agreement between all ranking models in this case study.

#### Significance of the spearman rank correlation coefficient

To statistically assess the level of conformity between the rankings generated by the WSM, WPM, and the combination of the WASPAS model, the Spearman Rank Correlation Coefficient is used. As WASPAS approach is a combination of both WSM and WPM by a convex combination parameter $\lambda$, it is necessary to check whether these various ranking mechanisms give similar results. In the absence of this validation, the reliability and stability of the hybrid framework might be doubted.

We quantitatively determine the monotonic consistency of the ranking lists by computing Spearman 0. The resultant correlation values ($\rho =0.9$ and $\rho =1$) show a very strong positive correlation between the results of the ranking. This indicates that the substitutes have almost the same ranking in the various assessment models, proving that the ultimate decision made is not sensitive to the aggregation mechanism. Thus, the significance of the Spearman analysis of this study is based on:

1. Checking ranking stability in additive, multiplicative, and combined WASPAS models.
2. Validating the soundness of the proposed hybrid approach in the case of methodological variation.
3. Giving statistical arguments in favor of the accuracy of the final decision result.
4. Increasing credibility of the experiment, particularly when it comes to a group decision-making context where there is uncertainty and experts are reluctant to contribute.

## Sensitivity analysis

A sensitivity analysis is carried out to examine how stable and reliable the decision outcomes obtained are. The details of this analysis are presented in Table 18, within the SWARA–WASPAS framework under CIFS conditions. The primary purpose of the analysis is to investigate how changes in the main input parameters influence the final ranking of alternatives. Specifically, once the attribute weights are determined using the SWARA method and the alternatives are evaluated through the WASPAS technique, sensitivity analysis is performed by systematically varying the key parameters. This process includes modifying the attribute weights derived from SWARA or adjusting the WASPAS trade-off parameter ${\boldsymbol{\lambda}}$, which controls the balance between the WSM and the WPM. To observe the impact of these variations on the ranking results, the ${\boldsymbol{\lambda}}$ parameter is typically varied from 0 to 1 with an increment of 0.1.

**Table 18:** Scores and rankings from the sensitivity analysis that are based on varying parameter values.

$\lambda$	${\mathcal{A}}_{1}$	${\mathcal{A}}_{2}$	${\mathcal{A}}_{3}$	${\mathcal{A}}_{4}$	${\mathcal{A}}_{5}$	**Ranking**	**Optimal**
${{\boldsymbol{\lambda}}}_{1}=0.1$	0.33	0.82	0.34	0.42	0.36	${\mathcal{A}}_{2}>{\mathcal{A}}_{4}>{\mathcal{A}}_{5}>{\mathcal{A}}_{3}>{\mathcal{A}}_{1}$	${\mathcal{A}}_{2}$
${{\boldsymbol{\lambda}}}_{2}=0.2$	0.34	0.82	0.35	0.44	0.37	${\mathcal{A}}_{2}>{\mathcal{A}}_{4}>{\mathcal{A}}_{5}>{\mathcal{A}}_{3}>{\mathcal{A}}_{1}$	${\mathcal{A}}_{2}$
${{\boldsymbol{\lambda}}}_{3}=0.3$	0.33	0.82	0.36	0.46	0.38	${\mathcal{A}}_{2}>{\mathcal{A}}_{4}>{\mathcal{A}}_{5}>{\mathcal{A}}_{3}>{\mathcal{A}}_{1}$	${\mathcal{A}}_{2}$
${{\boldsymbol{\lambda}}}_{4}=0.4$	0.35	0.82	0.37	0.48	0.38	${\mathcal{A}}_{2}>{\mathcal{A}}_{4}>{\mathcal{A}}_{5}>{\mathcal{A}}_{3}>{\mathcal{A}}_{1}$	${\mathcal{A}}_{2}$
${{\boldsymbol{\lambda}}}_{5}=0.5$	0.35	0.83	0.38	0.49	0.39	${\mathcal{A}}_{2}>{\mathcal{A}}_{4}>{\mathcal{A}}_{5}>{\mathcal{A}}_{3}>{\mathcal{A}}_{1}$	${\mathcal{A}}_{2}$
${{\boldsymbol{\lambda}}}_{6}=0.6$	0.35	0.83	0.39	0.51	0.40	${\mathcal{A}}_{2}>{\mathcal{A}}_{4}>{\mathcal{A}}_{5}>{\mathcal{A}}_{3}>{\mathcal{A}}_{1}$	${\mathcal{A}}_{2}$
${{\boldsymbol{\lambda}}}_{7}=0.7$	0.36	0.83	0.39	0.53	0.41	${\mathcal{A}}_{2}>{\mathcal{A}}_{4}>{\mathcal{A}}_{5}>{\mathcal{A}}_{3}>{\mathcal{A}}_{1}$	${\mathcal{A}}_{2}$
${{\boldsymbol{\lambda}}}_{8}=0.8$	0.36	0.83	0.40	0.55	0.42	${\mathcal{A}}_{2}>{\mathcal{A}}_{4}>{\mathcal{A}}_{5}>{\mathcal{A}}_{3}>{\mathcal{A}}_{1}$	${\mathcal{A}}_{2}$
${{\boldsymbol{\lambda}}}_{9}=0.9$	0.37	0.83	0.41	0.57	0.43	${\mathcal{A}}_{2}>{\mathcal{A}}_{4}>{\mathcal{A}}_{5}>{\mathcal{A}}_{3}>{\mathcal{A}}_{1}$	${\mathcal{A}}_{2}$

**Fig. 3:**
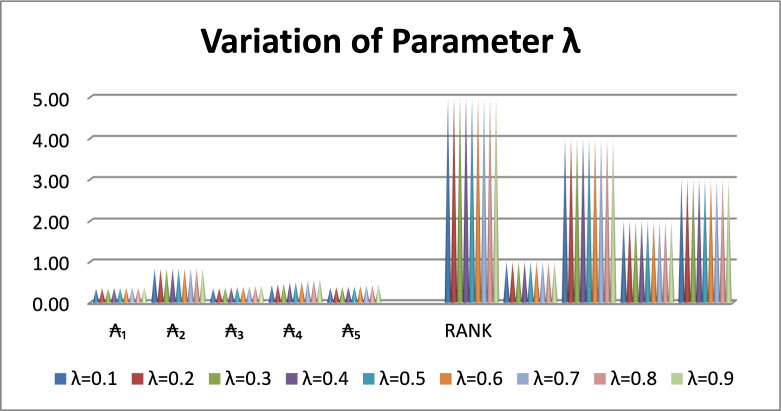
Sensitivity graph regarding changes in input parameters.

The ranking of the alternatives remains unchanged and ${\mathcal{A}}_{2}$ consistently achieving the highest performance score. At these specific values, ${\mathcal{A}}_{2}$ emerges as the most preferred alternative. The observed stability of the proposed SWARA–WASPAS approach demonstrates its strong robustness to variations in the balancing parameter between the WSM and the WPM. Table 18 highlights the significance of the sensitivity analysis in evaluating the reliability and consistency of the proposed decision-making framework. The results clearly show that ₂ outperforms the other alternatives across all considered parameter values, thereby supporting the validity of the analytical results. Furthermore, Fig. 3 provides a graphical illustration of the influence of different ${\boldsymbol{\lambda}}$ values on the performance scores of the alternatives, indicating that changes in the parameter have only a minimal effect on the final rankings. This visual evidence further confirms the stability and reliability of the proposed method.

## Comparative analysis

The suggested SWARA method and WASPAS addition to the CIFS framework are also a very promising improvement compared to the current MAGDM methods. In contrast to traditional fuzzy decision-making frameworks, where membership information, which is a single value, is used, the CIFS framework uses membership, non-membership, hesitation, and circular radius parameters to represent evaluation information. Such richer representation allows the decision-maker to state both the strength of the preferences as well as the dispersion and uncertainty of the judgments, which is specifically significant in complex and dynamic areas like career planning and job advice.

Furthermore, robustness analysis indicates that the hybrid SWARA–WASPAS structure remains stable under parameter variations and divergent expert opinions, outperforming classical fuzzy and non-circular models. These advantages make the proposed approach highly suitable for safety–critical and large-scale decision-making environments. A detailed summary of the results is provided in Table 19.

**Table 19:** Comparative characteristics of decision-making methods.

Characteristics	Traditional SWARA/WASPAS	Fuzzy SWARA/WASPAS	Proposed CIFS-SWARA-WASPAS
Uncertainty handling	Uses crisp numerical values; unable to represent uncertainty or hesitation	Handles linguistic terms using fuzzy numbers but assumes precise membership values	Models uncertainty through CIFS, explicitly capturing hesitation, ambiguity, and confidence levels
Representation of expert judgments	Fixed and deterministic judgments	Partially flexible through fuzzy membership	Highly flexible; represents membership and non-membership with radius
Attribute weight determination	Crisp or fixed fuzzy weights; limited realism	Fuzzy weights reflect uncertainty to some extent	CIFS-based SWARA allows intuitive and realistic weight assignment under uncertainty
Alternative evaluation	Deterministic scoring using WSM and WPM	Fuzzy scoring improves adaptability	CIFS-based WASPAS integrates uncertainty into scoring and ranking processes
Ranking stability	Sensitive to inconsistent or incomplete data	Moderately stable	Highly stable due to explicit modeling of hesitation and uncertainty
Handling of group decision-making	Limited ability to handle conflicting expert opinions	Improved but still sensitive to inconsistency	Effectively aggregates diverse and contradictory expert opinions
Robustness	Low robustness in uncertain environments	Moderate robustness	High robustness, confirmed through sensitivity analysis and rank correlation
Compatibility with existing models	Baseline approach	Compatible with traditional methods	Fully compatible and extendable from both traditional and fuzzy SWARA–WASPAS
Validation support	Usually lacks statistical validation	Limited validation	Strong validation using a case study, Spearman’s rank correlation, and sensitivity analysis
Practical applicability	Suitable for simple, well-defined problems	Applicable to moderately uncertain problems	Highly suitable for complex, real-world MAGDM problems under uncertainty

As can be seen in Table 19, the CIFS-based SWARA-WASPAS approach is better than the traditional and fuzzy counterparts as it provides better uncertainty modeling, robustness, and reliability. Its ability to fit within the current decision-making frameworks and its high levels of validation also justify its quality and high applicability in complex contexts of group decision-making with multiple attributes.

### Discussion

The findings of this work evidenced that the developed CIFS-SWARA-WASPAS framework can be significantly better than the traditional SWARA-WASPAS and fuzzy SWARA-WASPAS approaches. The CIFS framework uses membership, non-membership, hesitation, and radius of membership and non-membership, unlike the single-value membership in conventional methods, which use fuzzy numbers, which allows much more effective representation of the expert judgments. This multidimensional modeling enables the decision-maker to describe how strong his or her preferences are, as well as the uncertainty or dispersion of his or her evaluation, which is especially crucial in the complex and dynamic field of career planning and job guidance. These results are consistent with the recent studies focusing on the significance of modeling hesitation and uncertainty in group decision-making with multiple attributes^40^. The hybrid SWARA-WASPAS model also illustrates high robustness to change of parameters and divergent views of the experts. This validates previous findings that weighting and ranking techniques are beneficial in increasing stability in uncertain decision-making situations^31^. Compared to classical fuzzy models, the CIFS-based one is more robust to inconsistent or incomplete data, in line with the opinion that the explicit use of hesitation and uncertainty enhances reliability and ranking stability.

Table 19 also demonstrates that CIFS-SWARA-WASPAS is more effective than traditional and fuzzy equivalents in various aspects, such as uncertainty management, expert judgment flexibility, strength, and value support. The framework is effective in combining conflicting expert ideas, and this is a weakness of traditional group decision-making processes. Close endorsement of findings by sensitivity analysis and Spearman rank correlation also indicates that the methodology is useful to real-life MAGDM issues. Altogether, the results indicate that the CIFS-SWARA-WASPAS framework is a reliable, flexible, and strong tool to be used in complex decision-making situations. The explicit and explicit modeling of uncertainty and hesitation, the approach not only increases the accuracy of the decisions but also is consistent with the modern trends in the fuzzy and hybrid MAGDM research.

## Conclusion

This paper established a holistic hybrid approach of decision making through the combination of CIFS with the SWARA and WASPAS models in addressing uncertainty, reluctance, and subjectivity in MAGDM problems successfully. The classic SWARA and WASPAS methods and fuzzy versions in particular are frequently limited by their use of fixed or point-valued representations, limiting their capability to represent real-world ambiguity and human cognition. The proposed framework allows the decision-makers to capture both degrees of membership and non-membership in circular areas by introducing the notion of CIFS, which will provide a more realistic and flexible way of modeling expert judgments.

The SWARA method of CIFS-based, based on using expert hesitation and uncertainty, was used successfully to obtain attribute weights, and the CIFS-based WASPAS technique offered a powerful tool in assessing and scoring alternatives and ranking them. The combination of weighted sum and weighted product models on the CIFS environment increased the accuracy and stability of ranking, even when there are incomplete or inconsistent expert opinions. The efficiency of the suggested approach was proven with the help of a real case study concentrated on the maximization of vocational training, career planning, and employment guidance programs. The findings reveal that “vocational training combined with basic career counseling” always tops the list of all the models. Moreover, the framework was found to be robust by using Spearman rank correlation analysis and sensitivity analysis that indicated strong agreement among the dissimilar ranking approaches and stability of rankings, respectively, in alteration of the WASPAS trade-off parameter. These validation findings prove that the proposed hybrid solution is reliable and consistent. The comparative analysis demonstrates that the proposed SWARA–WASPAS method outperforms traditional and fuzzy variants by offering superior uncertainty modeling and greater robustness.

### Results and analysis

More clearly, immediately after presenting the research questions in the Introduction Section "Introduction". The results are now systematically discussed in Section "Case study" according to those sub-questions. The findings related to the integration of CIFS with SWARA and WASPAS are presented first, addressing how uncertainty, hesitation, and ambiguity are modeled. Then, the attribute weight determination results, Table 4 to Table 9, and alternative ranking outcomes, Table 14 to Table 15, are organized to correspond to the research question concerning the plausibility and realism of weights and rankings. Furthermore, the robustness and stability issue is addressed in Section "Sensitivity analysis", where the Spearman rank correlation analysis and sensitivity analysis Table 16 to Table 18, are discussed as direct responses to the research question on ranking consistency under parameter variation and expert inconsistency.

### Limitation and future direction

Although the suggested CIFS-based SWARA-WASPAS framework proves to be quite effective when it comes to addressing the issue of uncertainty and expert hesitation, there are still some weaknesses. The model mainly relies on linguistic assessments by experts and predetermined features, which can become subjective and limiting in the case of application to large-scale or dynamically changing decision making scenario. In addition, the recent application is narrowed to CIFS, and other superior fuzzy representations, including circular q-rung orthopair FS, circular spherical FS, or bipolar fuzzy models, are yet to be studied in this framework. Future studies can improve the suggested method by incorporating data-oriented methodologies, including machine learning models of automatic weight estimation and artificial intelligence systems of real-time data collection. In addition, it can be further hybridized with other sophisticated decision-making methods, including DEMATEL, TOPSIS, VIKOR, or AROMAN, which might be additional means to improve the level of analysis and predictability of the framework.

The primary objective of this study was to develop a robust methodological framework capable of handling uncertainty, hesitation, and subjective expert judgments in MAGDM problems. To ensure internal validity and methodological soundness, we incorporated a structured expert evaluation process, Spearman rank correlation analysis to verify ranking consistency, and sensitivity analysis by varying the WASPAS trade-off parameter ${\boldsymbol{\lambda}}$. The stability of rankings, particularly the consistent top performance of “vocational training combined with basic career counseling,” demonstrates the robustness of the proposed work.

## Data Availability

The datasets used and/or analyzed during the current study are available from the corresponding author upon reasonable request.

## Code availability

The computational framework and calculation workflow used to generate the results are provided as a structured Microsoft Excel file and are publicly available via Zenodo at 10.5281/zenodo.19054983. The file contains all intermediate steps, formula-based computations, and ranking procedures required to reproduce the analysis.
